# Lactic Acid Fermentation Is Required for NLRP3 Inflammasome Activation

**DOI:** 10.3389/fimmu.2021.630380

**Published:** 2021-03-29

**Authors:** Hsin-Chung Lin, Yu-Jen Chen, Yau-Huei Wei, Hsin-An Lin, Chien-Chou Chen, Tze-Fan Liu, Yi-Lin Hsieh, Kuo-Yang Huang, Kuan-Hung Lin, Hsueh-Hsiao Wang, Lih-Chyang Chen

**Affiliations:** ^1^Graduate Institute of Medical Sciences, National Defense Medical Center, Taipei, Taiwan; ^2^Division of Clinical Pathology, Department of Pathology, Tri-Service General Hospital, Taipei, Taiwan; ^3^Department of Radiation Oncology, MacKay Memorial Hospital, New Taipei City, Taiwan; ^4^Department of Medical Research, MacKay Memorial Hospital, New Taipei City, Taiwan; ^5^Department of Nursing, MacKay Junior College of Medicine, Nursing, and Management, Taipei, Taiwan; ^6^Center for Mitochondrial Medicine and Free Radical Research, Changhua Christian Hospital, Changhua, Taiwan; ^7^Department of Medicine, Tri-Service General Hospital SongShan Branch, Taipei, Taiwan; ^8^Department of Medicine, MacKay Medical College, New Taipei City, Taiwan; ^9^Graduate Institute of Pathology and Parasitology, National Defense Medical Center, Taipei, Taiwan; ^10^Institute of Biomedical Sciences, MacKay Medical College, New Taipei City, Taiwan

**Keywords:** glycolysis, lactic acid fermentation, pyruvate oxidation, NLRP3 inflammasome, inflammation

## Abstract

Activation of the Nod-like receptor 3 (NLRP3) inflammasome is important for activation of innate immune responses, but improper and excessive activation can cause inflammatory disease. We previously showed that glycolysis, a metabolic pathway that converts glucose into pyruvate, is essential for NLRP3 inflammasome activation in macrophages. Here, we investigated the role of metabolic pathways downstream glycolysis – lactic acid fermentation and pyruvate oxidation—in activation of the NLRP3 inflammasome. Using pharmacological or genetic approaches, we show that decreasing lactic acid fermentation by inhibiting lactate dehydrogenase reduced caspase-1 activation and IL-1β maturation in response to various NLRP3 inflammasome agonists such as nigericin, ATP, monosodium urate (MSU) crystals, or alum, indicating that lactic acid fermentation is required for NLRP3 inflammasome activation. Inhibition of lactate dehydrogenase with GSK2837808A reduced lactate production and activity of the NLRP3 inflammasome regulator, phosphorylated protein kinase R (PKR), but did not reduce the common trigger of NLRP3 inflammasome, potassium efflux, or reactive oxygen species (ROS) production. By contrast, decreasing the activity of pyruvate oxidation by depletion of either mitochondrial pyruvate carrier 2 (MPC2) or pyruvate dehydrogenase E1 subunit alpha 1 (PDHA1) enhanced NLRP3 inflammasome activation, suggesting that inhibition of mitochondrial pyruvate transport enhanced lactic acid fermentation. Moreover, treatment with GSK2837808A reduced MSU-mediated peritonitis in mice, a disease model used for studying the consequences of NLRP3 inflammasome activation. Our results suggest that lactic acid fermentation is important for NLRP3 inflammasome activation, while pyruvate oxidation is not. Thus, reprograming pyruvate metabolism in mitochondria and in the cytoplasm should be considered as a novel strategy for the treatment of NLRP3 inflammasome-associated diseases.

## Introduction

The Nod-like receptor 3 (NLRP3) inflammasome is a multiprotein complex that is composed of a cytosolic pattern recognition receptor, NLRP3, along with the adaptor protein, apoptosis associated speck-like protein containing a CARD (ASC), and the cysteine-aspartic acid protease-1 (caspase-1) (1). NLRP3 inflammasome formation is initiated by the oligomerization of NLRP3, which recruits ASC to activate caspase-1, and in turn mediates the cleavage of pro-interleukin 1 beta (pro-IL-1β) or pro-interleukin 18 (pro-IL-18) to the mature proinflammatory cytokines, IL-1β and IL-18 (2). NLRP3 is an important receptor for inflammasome activation in response to some damage-associated molecular patterns (DAMPs) and pathogen-associated molecular patterns (PAMPs), such as monosodium urate (MSU) crystal, an etiological agent of gout (3), and nigericin, a pore-forming toxin derived from *Streptomyces hygroscopicus* (4). NLRP3 deficiency increases susceptibility to microbial infection in mice (5). In addition, increasing evidence suggests that excessive and improper inflammasome activation is responsible for the pathogenesis of several inflammation-associated diseases, including gout (3), type 2 diabetes (6), atherosclerosis (7), rheumatoid arthritis (8), septic shock (9), cryopyrin-associated periodic syndrome (10), Alzheimer's disease (11), and cancer (12).

NLRP3 inflammasome activation includes multiple upstream signals, such as reactive oxygen species (ROS) production and potassium efflux (13). Mitochondria are the major source of cellular ROS, which are generated as by-products of respiration and oxidative phosphorylation (OXPHOS). Blocking the electron transport chain increases mitochondrial ROS (mtROS) production. This increase of mtROS production is sufficient for activation of the NLRP3 inflammasome, suggesting that mtROS is an activator of the NLRP3 inflammasome (14). Consistent with this hypothesis, nigericin induces mtROS production and activates the NLRP3 inflammasome in macrophages, whereas treatment with a mitochondria-specific ROS scavenger, mito-TEMPO, can inhibit NLRP3 inflammasome activation (15). On the other hand, potassium efflux is suggested as the common trigger of NLRP3 inflammasome. After stimulation with nigericin or MSU, potassium efflux was induced immediately and was followed by NLRP3 inflammasome activation (4). In addition, high extracellular concentrations of potassium prevent NLRP3 inflammasome activation, whereas low intracellular concentrations of potassium are sufficient to activate the NLRP3 inflammasome in macrophages (4, 16). Because mtROS and potassium ions are unlikely to interact directly with the NLRP3 protein, the underlying mechanisms of NLRP3 inflammasome regulation remains to be elucidated.

Glycolysis is a critical pathway in cellular glucose metabolism that provides intermediates for energy generation. Glycolysis metabolizes one molecule of glucose to two molecules of pyruvate, with the concomitant net production of two molecules of ATP in the cytoplasm. Pyruvate can be further processed anaerobically by lactate dehydrogenase (LDH) to lactate (termed lactic acid fermentation) or be further transported into mitochondria by mitochondrial pyruvate carrier (MPC) and then processed aerobically to acetyl-CoA by pyruvate dehydrogenase (PDH) (termed pyruvate oxidation). Acetyl-CoA is then used to generate much more ATP through the tricarboxylic acid (TCA) cycle and OXPHOS.

Emerging evidence indicates that glycolysis is involved in NLRP3 inflammasome activation in macrophages (17–19). We previously showed that the levels of glycolysis and OXPHOS modulate NLRP3 inflammasome activation in macrophages (17). Production of mtROS regulated by hexokinase (HK) was suggested to be important for glycolysis-dependent NLRP3 inflammasome activation (18), and lactate-dependent PKR phosphorylation regulated by pyruvate kinase muscle isoenzyme 2 (PKM2) was needed (19, 20). Since glycolysis is branched to pyruvate oxidation-driven OXPHOS in mitochondria or lactic acid fermentation in the cytoplasm, the mechanisms underlying glycolysis-dependent activation of the NLRP3 inflammasome are still poorly understood.

In this study, we discovered that lactic acid fermentation is important for NLRP3 inflammasome activation, but pyruvate oxidation is not. In response to cell stimulation with NLRP3 inflammasome agonist, lactic acid fermentation is activated to produce lactate, which induces PKR phosphorylation and consequently activates the NLRP3 inflammasome; which is positively regulated by inhibition of pyruvate oxidation. Importantly, treatment with GSK2837808A, a LDH inhibitor, could efficiently protect mice from MSU-mediated peritonitis. Together, our findings provide new insights into the role of pyruvate metabolism in NLRP3 inflammasome activation and suggest that reprogramming of pyruvate metabolism in mitochondria and cytoplasm should be considered as a therapeutic target for treatment of NLRP3 inflammasome-associated diseases.

## Materials and Methods

### Reagents, Antibodies, and Plasmids

PMA, ATP, 2DG, nigericin, and PhosSTOP were purchased from Sigma/Aldrich Chemical Co. (St. Louis, MO). MSU and alum were purchased from InvivoGen. GSK2837808A, Z-VAD-FMK, and C16 were purchased from MedChemExpress. Anti-CD45-APC and anti-CD11b-BV605 were purchased from BioLegend. Anti-Ly6G-PE was obtained from BD Bioscience.

### Animal Experiments

Mouse experiments were performed under the ethical approval by the Institutional Animal Care and User Committee of MacKay Medical College (approval number: A1080017). Female C57BL/6J mice were used at 8 weeks of age. For MSU-induced peritonitis, mice were intraperitoneally treated with dimethyl sulfoxide or GSK2837808A (20 mg per kg body weight) at 30 min before challenge with MSU (1 mg). At 4 h after MSU treatment, peritoneal lavage fluids were collected. The peritoneal cells were subjected to antibody staining and the number of neutrophils (CD45^+^/CD11b^+^/Ly6G^+^) were analyzed on a CytoFLEX S flow cytometer (Beckman Coulter).

### Cell Culture

Mouse bone marrow-derived macrophages (BMDMs) were generated as described previously (21). BMDMs were prepared from bone marrow cells, which were collected from the tibias and femurs of C57BL/6J mice by flushing with cold PBS using a 25-G needle. The cells were cultured in DMEM medium supplemented with 10% FCS and 10 ng/ml M-CSF (PeproTech) for 8 days. The human leukemia monocytic cell line, THP-1, was obtained from the Biosource Collection and Research Center, Food Industry Research and Development Institute (Hsinchu, Taiwan) and maintained in RPMI as described previously (22). For macrophage differentiation, THP-1 cells were stimulated with 200 nM PMA for 16 h as described previously (17). For inflammasome stimulation, the cells were treated with 10 μM nigericin for 45 min, 5 mM ATP, 200 μg/ml MSU, or 200 μg/ml alum for 4 h. For lactic acid fermentation inhibition and caspase inhibition, GSK2837808A (10 μM), or Z-VAD-FMK (20 μM) was applied for 1 h prior to inflammasome stimulation.

### Assays for Mitochondrial Respiration and Glycolysis

Approximately 5 × 10^4^ cells/well THP-1 cells were plated on the cell culture microplates of an XF24 extracellular flux analyzer (Seahorse Bioscience, Billerica, MA, USA). The extracellular acidification rate (ECAR) and oxygen consumption rate (OCR), parameters of glycolytic flux and mitochondrial respiration, respectively, were continuously measured on the Seahorse XF24 according to the manufacturer's instructions as described previously (17).

### Immunoblot Analysis

Cell lysates were prepared using RIPA buffer (50 mM Tris-HCl, pH 7.5, 150 mM NaCl, 10 mM MgCl_2_, 1 mM EDTA, 1% Igepal CA-630) with a protease inhibitor cocktail (4.76 μg/ml leupeptin, 3.25 μg/ml aprotinin, 0.69 μg/ml pepstatin and 1 mM phenylmethylsulfonyl fluoride) and a phosphatase inhibitor cocktail PhosSTOP on ice for 30 min as described previously (17). To prepare the protein samples from the culture supernatants, culture supernatants were collected, mixed with one tenth volume of 100% (wt/vol) trichloroacetic acid, and incubated for 10 min at 4°C for precipitating the proteins. 20 μg of cell lysate samples or the half volume of culture supernatant samples were resolved by 12–15% SDS/PAGE and transferred to PVDF membranes (Millipore). After blocking with 5% nonfat dry milk in TBS-Tween 20, membranes were incubated with the indicated primary antibodies overnight at 4°C, and then with an HRP-conjugated secondary antibody for 1 h at room temperature. The primary antibodies included anti-GAPDH (Santa Cruz #sc-32233, 1:10,000), anti-ASC (Santa Cruz #sc-22514, 1:1,500), anti-IL-1β (Santa Cruz #sc-32294, 1:1,500), anti-procaspase-1 (Cell Signaling #2225, 1:1,000), anti-cleaved caspase-1 (Cell Signaling #3866, 1:1,000), anti-LDHA (Cell Signaling #3582, 1:15,000), and anti-MPC2 (Cell Signaling #46141, 1:1,000), anti-PKR (Abcam #ab226819, 1:1,000), anti-phosphorylated PKR (Merck Millipore #07-886, 1:2,000), anti-NLRP3 (Adipogen #AG-20B-0014-C100, 1:1,000), and anti-PDHA1 (Proteintech #18068-1-AP, 1:1,000). The immunoreactive bands were detected using an enhanced chemiluminescence system (Amersham Pharmacia Biotech, AB, Uppsala, Sweden). Immunoblot images were quantified with the ImageJ software.

### RNA Interference

The dsRNA duplexes were transfected into cells using Lipofectamine 2000 reagent (Invitrogen), as previously described (23). THP-1-derived macrophages were transfected with 50 nmol/l dsRNA duplexes and 2 μl/ml Lipofectamine 2000 reagents according to the manufacturer's protocol. At 6 h post-transfection, the siRNA containing medium was replaced with fresh complete medium. For efficient knockdown, the cells were incubated for 2 days. The reagent used to target LDHA and purchased from Dharmacon was a 19-bp RNA duplexes: 5′- GGCAA AGACU AUAAU GUAA−3′. The reagent used to target MPC2 and purchased from Dharmacon included three 19-bp RNA duplexes: 5′- GGGUU UAUUU GGUCA AGAU−3′ (#17), 5′- CCAUU GGGAC CUAGU UUAU−3′ (#19), and 5′- AGAAA UUGAG GCCGU UGUA−3′ (#20). The reagent used to target PDHA1 and purchased from Ambion included two 19-bp RNA duplexes: 5′- GACUU ACCGU UACCA CGGA−3′ (#s10243) and 5′- CUGUG AGAAU AAUCG CUAU−3′ (#s10245).

### Flow Cytometric Isolation of ASC-mCherry Speck-Forming Cells

The ASC-mCherry-expressing THP-1-derived macrophages stimulated with or without nigericin for 1 h were analyzed by FACSAria cell sorter (BD Biosciences) using the 561 nm laser and 610/20 nm filter. After nigericin stimulation, the ASC-mCherry speck-positive and negative cells were sorted using a FACSAria cell sorter (BD Biosciences) as described previously (21, 24). The cells with or without ASC-mCherry speck formation was defined as low or high ASC-mCherry pulse width to area profile (W:A), respectively, and sorted for the presence/absence of ASC-mCherry specks.

### Lactate Assay

Lactate in culture medium was measured with the Lactate Assay Kit (Cell Biolabs, #MET-5012) according to the manufacturer's instructions.

### Potassium Efflux Assay

Intracellular potassium ion was measured using Asante Potassium Green-4 (APG-4) (Interchim). The cells were incubated with 1 μM APG-4 at 37 °C for 45 min, and then stimulated with nigericin as indicated and analyzed immediately by flow cytometry.

### IL-1β Enzyme-Linked Immunosorbent Assay

IL-1β concentrations of cell culture supernatants and mouse peritoneal lavage fluids were measured by using human IL-1β ELISA kit and mouse IL-1β ELISA kit that were purchased from Thermo Fischer Scientific.

### Mitochondrial and Cellular ROS Measurements

Mitochondrial and cellular ROS were measured using MitoSox Red and H_2_-DCFDA (Thermo Fischer Scientific), respectively. The cells were incubated with 1 μM MitoSox Red or 0.3 μM H_2_-DCFDA at 37°C for 30 min, and then analyzed by flow cytometry.

### Statistical Analysis

All statistical analyses were performed with the Student's *t*-test as indicated or ANOVA test followed by Bonferroni *post hoc* test in SPSS as described previously (25). Differences were considered significant at *p* < 0.05.

## Results

### Lactic Acid Fermentation Is Essential for NLRP3 Inflammasome Activation

**Figure 1:**
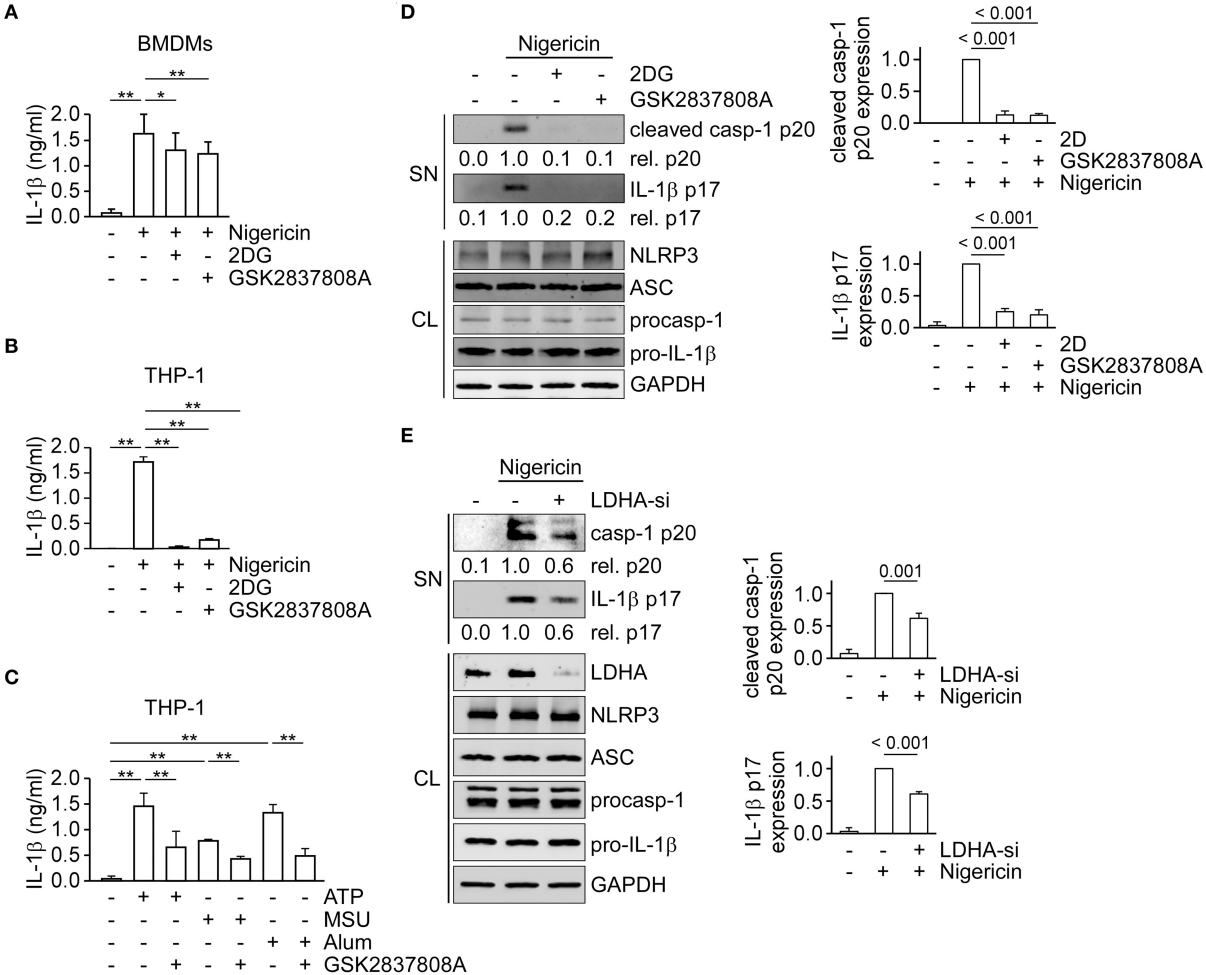
Inhibition of lactate dehydrogenase prevents NLRP3 inflammasome activation. **(A)** ELISA was used to determine the IL-1β levels in culture supernatants of mouse bone marrow-derived macrophages (BMDMs) that had been pretreated with 2DG or GSK2837808A for 1 h, followed by stimulation with nigericin for 45 min. **(B)** Determination by ELISA of IL-1β in the culture supernatant of THP-1-derived macrophages that had been pretreated with 2DG or GSK2837808A for 1 h, followed by stimulation with nigericin for 45 min. **(C)** Determination by ELISA of IL-1β in the culture supernatant of THP-1-derived macrophages that had been pretreated with GSK2837808A for 1 h, followed by stimulation with ATP, MSU, or alum for 4 h. **(D)** Immunoblot analysis of NLRP3 inflammasome molecules in culture supernatants (SN) and cell lysates (CL) of THP-1-derived macrophages that had been treated with or without 2DG or GSK2837808A for 1 h and then stimulated with nigericin for 45 min. The densitometric analysis of caspase-1 (p20) and IL-1β (p17) was shown in the right panels. **(E)** Immunoblot analysis of NLRP3 inflammasome molecules in cell supernatants and cell lysates of THP-1-derived macrophages treated with LDHA- or negative control- siRNA, and then stimulated with nigericin for 45 min. The densitometric analysis of caspase-1 (p20) and IL-1β (p17) was shown in the right panels. *P < 0.05; **P < 0.01. All results are presented as the mean ± SD of data from three independent experiments (*n* = 3 per group), and were analyzed with the ANOVA test followed by *post hoc* test.

Reports from our group and others showed that glycolysis is essential for NLRP3 inflammasome activation (17–19, 26); therefore, we hypothesized that the metabolic pathways downstream from glycolysis—lactic acid fermentation and/or pyruvate oxidation—should also be considered in activation of the NLRP3 inflammasome. Thus, we first examined the effect of inhibition of lactic acid fermentation on NLRP3 inflammasome dependent IL-1β secretion in mouse bone marrow-derived macrophages (BMDMs) (Figure 1A) or THP-1-derived macrophages (Figure 1B). We found that IL-1β secretion in response to nigericin was significantly inhibited by the glycolysis inhibitor 2-deoxyglucose (2DG) or LDH inhibitor GSK2837808A. To examine if the LDH-mediated mechanism was a common pathway of NLRP3 inflammasome regulation, we further assessed the effect of GSK2837808A on IL-1β secretion in response to other NLRP3 inflammasome agonists: ATP, MSU or alum. Consistently, GSK2837808A inhibited ATP-, MSU- or alum-induced IL-1β secretion (Figure 1C). Finally, to determine whether lactic acid fermentation was involved in NLRP3 inflammasome activation, we assessed the effect of LDH inhibition (with pharmacological agents or by genetic depletion) on the activation of caspase-1 and maturation of IL-1β in THP-1-derived macrophages stimulated with nigericin. The levels of mature IL-1β p17 and active caspase-1 (as assessed by the level of caspase-1 p20) were reduced in cells treated with GSK2837808A (Figure 1D) or lactate dehydrogenase A (LDHA)-specific small interfering RNA (siRNA) (Figure 1E), compared with the corresponding control cells, while NLRP3, ASC, pro-caspase-1 and pro-IL-1β levels were unchanged. These results suggest that lactic acid fermentation is a common mechanism for NLRP3 inflammasome activation.

### Lactic Acid Fermentation Regulates NLRP3 Inflammasome Activation Through PKR Phosphorylation

**Figure 2:**
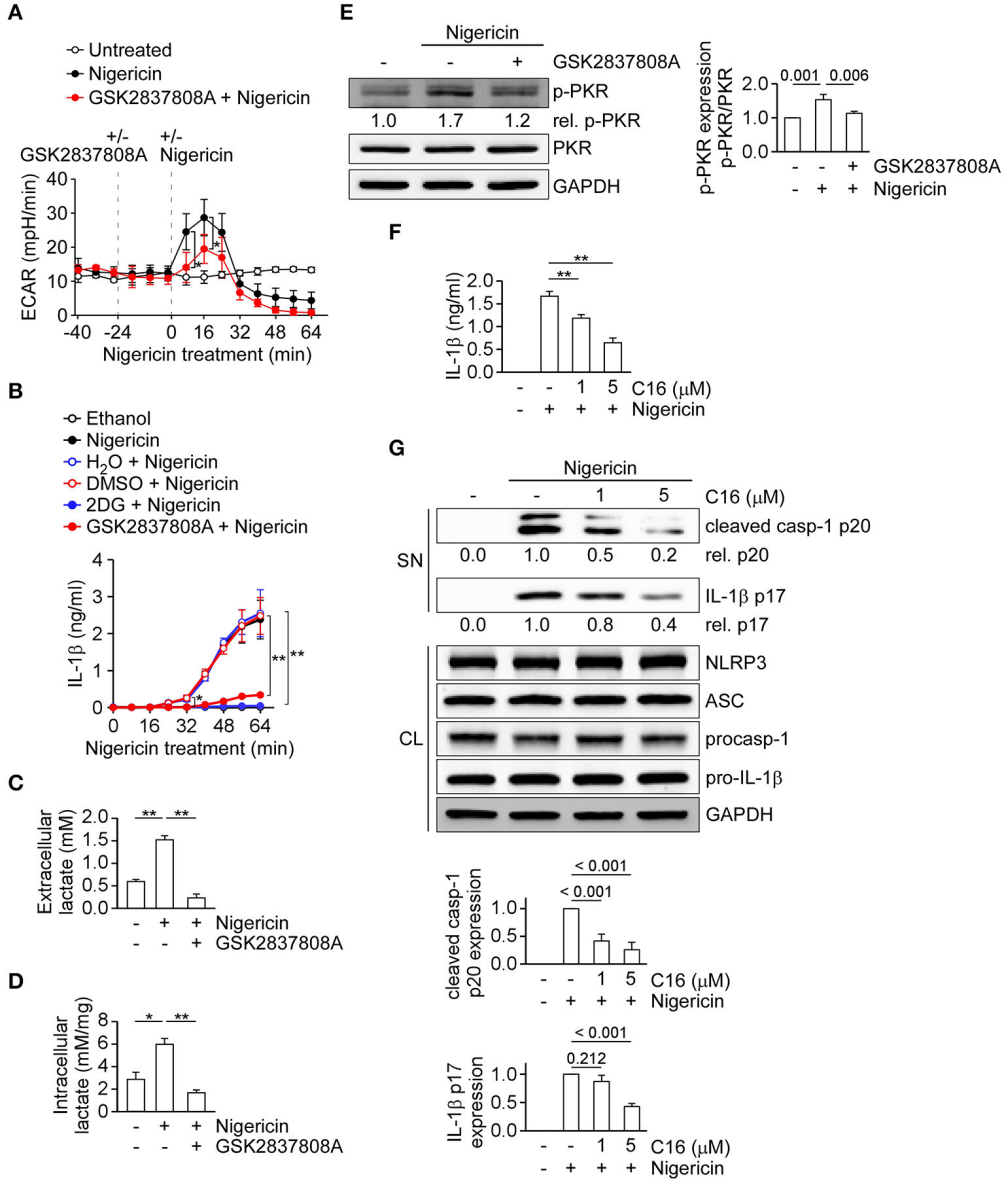
Inhibition of lactate dehydrogenase prevents lactate production and PKR phosphorylation upon NLRP3 inflammasome activation. **(A)** ECAR of THP-1-derived macrophages under sequential treatment (dotted vertical lines) with or without GSK2837808A and treated with or without nigericin. **(B)** IL-1β levels in the culture supernatant of THP-1-derived macrophages that had been pretreated with 2DG or GSK2837808A for 1 h, followed by stimulation with nigericin for 0 to 64 min. **(C)** The amount of extracellular lactate in the culture supernatant of THP-1-derived macrophages that had been pretreated with GSK2837808A for 1 h, followed by stimulation with nigericin for 45 min. **(D)** Lactate levels in the cell lysates of THP-1-derived macrophages that had been pretreated with GSK2837808A for 1 h, followed by stimulation with nigericin for 45 min. The lactate levels were calculated by normalization of lactate concentration with the protein levels of cell lysates. **(E)** Immunoblot analysis of phosphorylated PKR (p-PKR) in THP-1-derived macrophages that had been pretreated with GSK2837808A for 1 h, followed by stimulation with nigericin for 45 min. The relative amounts of p-PKR were calculated by normalization with total PKR, and the resulting values from the unstimulated control cells were set as 1.0. The densitometric analysis of p-PKR was shown in the right panel. **(F)** IL-1β levels in the culture supernatant of THP-1-derived macrophages that had been pretreated with C16 for 1 h, followed by stimulation with nigericin for 45 min. **(G)** Immunoblot analysis of NLRP3 inflammasome molecules in culture supernatants (SN) and cell lysates (CL) of THP-1-derived macrophages that had been treated with or without C16 for 1 h and then stimulated with nigericin for 45 min. The densitometric analysis of caspase-1 (p20) and IL-1β (p17) was shown in the bottom panel. *P < 0.05; **P < 0.01. All results are presented as the mean ± SD of the data from three independent experiments (*n* = 3 per group), and were analyzed with the ANOVA test followed by *post hoc* test.

To understand how lactic acid fermentation may regulate the NLRP3 inflammasome, we examined if lactic acid fermentation was stimulated when the NLRP3 inflammasome was activated. We measured the ECAR, an indicator of lactic acid fermentation, in THP-1-derived macrophages. We found that the ECAR values were immediately increased after nigericin stimulation at around 8 min and peaked at around 16 min, compared with untreated control cells, but were decreased by GSK2837808A pretreatment (Figure 2A). To study the correlation of lactate production and NLRP3 inflammasome activation, we analyzed the time point of nigericin-induced NLRP3 inflammasome activation by assessing IL-1β secretion in the presence or absence of GSK2837808A, 2DG, or the relevant solvent (Figure 2B). Nigericin started to induce IL-1β secretion at around 32 min, while the solvent control ethanol did not. Nigericin-induced IL-1β secretion was inhibited by GSK2837808A or 2DG, but not the relevant control DMSO or H_2_O. Upon nigericin stimulation, the induction of lactate production at 8 min was earlier than NLRP3 inflammasome activation at 32 min, suggesting that lactate production contributed to NLRP3 inflammasome activation. Therefore, we examined if lactate, the product of lactic acid fermentation, was induced upon NLRP3 inflammasome activation by measuring the extracellular and intracellular lactate concentrations in THP-1-derived macrophages. Consistent with the increase of ECAR by nigericin stimulation (Figure 2A), the levels of extracellular and intracellular lactate concentrations were increased by nigericin stimulation, compared with untreated control cells, but inhibited by GSK2837808A pretreatment (Figures 2C,D). As it was reported that lactate treatment can induce phosphorylation of PKR (19), which is a regulator of the NLRP3 inflammasome (20), we further examined if lactic acid fermentation regulates the NLRP3 inflammasome via PKR phosphorylation. As shown in Figure 2E, the levels of phosphorylated PKR were increased by nigericin stimulation but inhibited by GSK2837808A. These results suggest that lactic acid fermentation regulates NLRP3 inflammasome activation via lactate-dependent PKR phosphorylation. Therefore, we further examined whether PKR regulates activation of the NLRP3 inflammasome in our cell model. Pretreatment of PKR inhibitor C16 significantly reduced the secretion of IL-1β (Figure 2F) and the expression of active caspase-1 p20 and mature IL-1β p17 (Figure 2G) in THP-1-derived macrophages upon nigericin stimulation, suggesting that PKR contributes to activation of the NLRP3 inflammasome. Together with the previous results that lactate production regulated PKR phosphorylation (Figure 2E) and NLRP3 inflammasome activation (Figure 1D), it was suggested that lactate production regulated NLRP3 inflammasome activation through PKR signaling, at least partially.

### Inhibition of the Pyruvate Oxidation Pathway Augments Lactate Production and Enhances NLRP3 Inflammasome Activation

**Figure 3:**
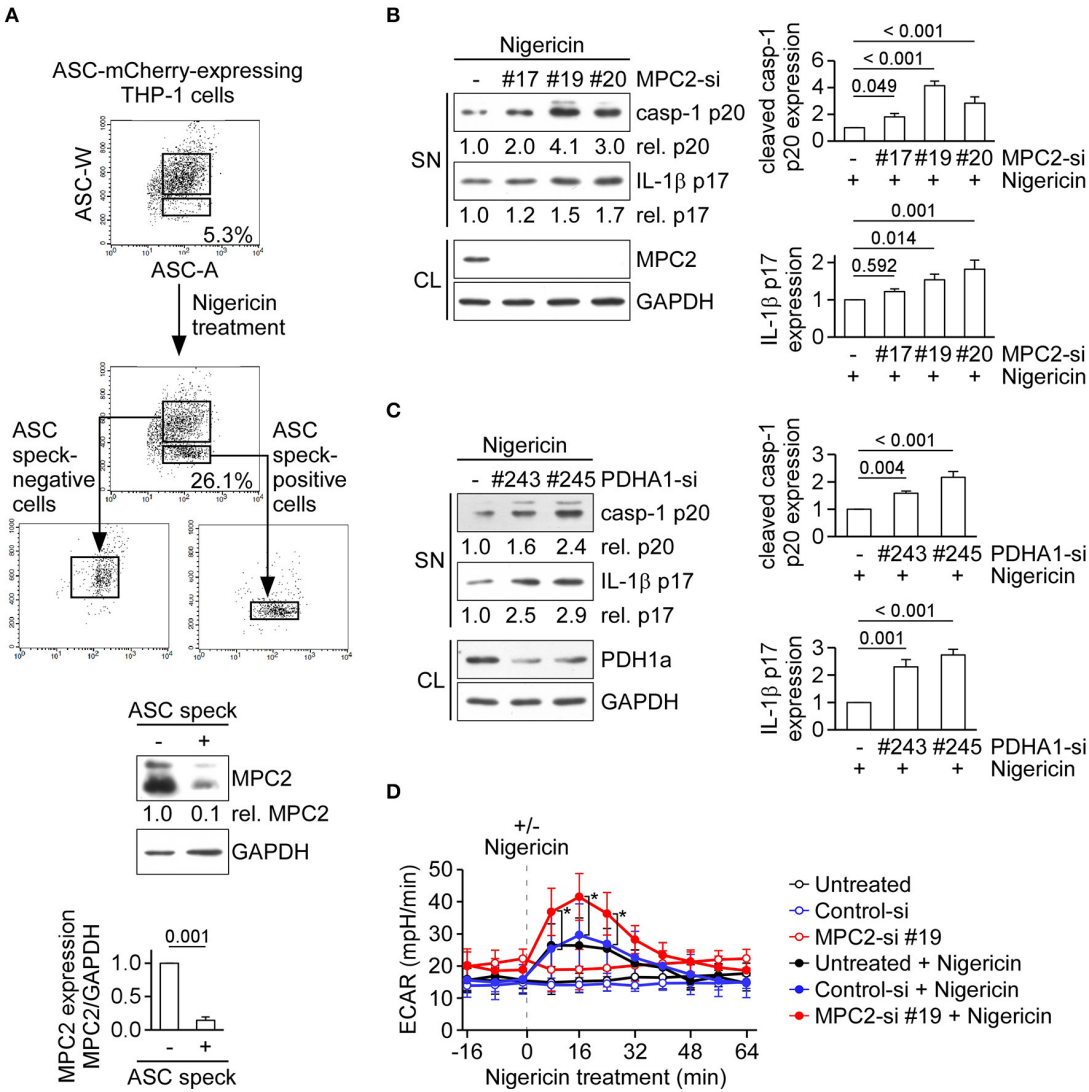
Inhibition of the pyruvate oxidation pathway augments lactate production and enhances NLRP3 inflammasome activation. **(A)** ASC-mCherry-expressing THP-1-derived macrophages were treated with nigericin for 1 h, ASC speck-containing cells were isolated by flow cytometry. Low and high ASC-W:ASC-A profiles were used, respectively, to indicate ASC speck-positive and -negative cells, and immunoblot was performed using anti-MPC2 and anti-GAPDH antibodies. The densitometric analysis of MPC2 was shown in the bottom panel. Results were analyzed with the Student's *t*-test. **(B)** Immunoblot analysis of NLRP3 inflammasome molecules in cell supernatants and cell lysates of THP-1-derived macrophages treated with MPC2- or negative control siRNA, and then stimulated with nigericin for 45 min. The densitometric analysis of caspase-1 (p20) and IL-1β (p17) was shown in the right panel. **(C)** Immunoblot analysis of NLRP3 inflammasome molecules in cell supernatants and cell lysates of THP-1-derived macrophages treated with PDHA1- or negative control siRNA, and then stimulated with nigericin for 45 min. The densitometric analysis of caspase-1 (p20) and IL-1β (p17) was shown in the right panel. **(D)** ECAR of THP-1-derived macrophages that had been pretreated with or without MPC2- or negative control siRNA, following stimulation with or without nigericin (dotted vertical lines). All results are presented as the mean ± SD of data from three independent experiments (*n* = 3 per group). Except for results of **(A)**, all results were analyzed with the ANOVA test followed by *post hoc* test. *P < 0.05.

Besides the conversion of pyruvate to lactate in cytoplasm, pyruvate can be transported to mitochondria by MPC and then converted into acetyl-CoA via pyruvate oxidation for pyruvate-driven respiration. MPC is composed of the MPC1/MPC2 heterodimer, which is responsible for transporting pyruvate into mitochondria (27). Here, we examined the role of MPC2 in NLRP3 inflammasome activation. During formation of the NLRP3 inflammasome complex, ASC molecules form a speck and oligomerize to recruit procaspase-1 prior to the activation of caspase-1. To quantify inflammasome formation in ASC speck-positive and -negative cells after stimulation, we carried out time-of-flight inflammasome evaluation analysis (24). In ASC-mCherry-expressing THP-1 cells treated with the NLRP3 agonist, nigericin, the percentage of ASC speck-containing cells was increased to 26.1%, compared with the 5.3% seen in untreated cells (Figure 3A, top). Notably, the level of MPC2 protein was 0.1-fold in ASC speck-containing cells relative to ASC speck-negative cells (Figure 3A, bottom). This suggests that there was a higher level of MPC2 protein in cells that failed to efficiently form the NLRP3 inflammasome complex. To evaluate whether MPC2 is involved in regulating the NLRP3 inflammasome activation, we analyzed changes in caspase-1 activation and IL-1β maturation in cells treated with nigericin. The levels of mature IL-1β p17 and active caspase-1 were increased in THP-1-derived macrophages treated with a MPC2-specific siRNA, compared with those treated with control siRNA (Figure 3B). As pyruvate oxidation is catalyzed by PDH, we further examined the role of PDH in NLRP3 inflammasome regulation. As shown in Figure 3C, the levels of mature IL-1β p17 and active caspase-1 were increased in THP-1-derived macrophages treated with a pyruvate dehydrogenase E1 subunit alpha 1 (PDHA1)-specific siRNA, compared with those treated with control siRNA. These results indicate that blocking the mitochondrial pyruvate metabolism pathway enhances activation of the NLRP3 inflammasome. We hypothesized that blocking of mitochondrial pyruvate metabolism enhanced NLRP3 inflammasome activation through altered pyruvate utilization in the cytoplasm. Thus, we assessed the effect of MPC2 knockdown on lactic acid fermentation by measuring ECAR. We found that the ECAR value increase by nigericin was further increased in THP-1-derived macrophages treated with MPC2 siRNA, compared with control siRNA (Figure 3D). These results suggest that inhibition of pyruvate oxidation pathway augments lactate production and thereby enhances NLRP3 inflammasome activation.

### Inhibition of Lactic Acid Fermentation Does Not Prevent ROS Production and Potassium Efflux Upon NLRP3 Inflammasome Activation

**Figure 4:**
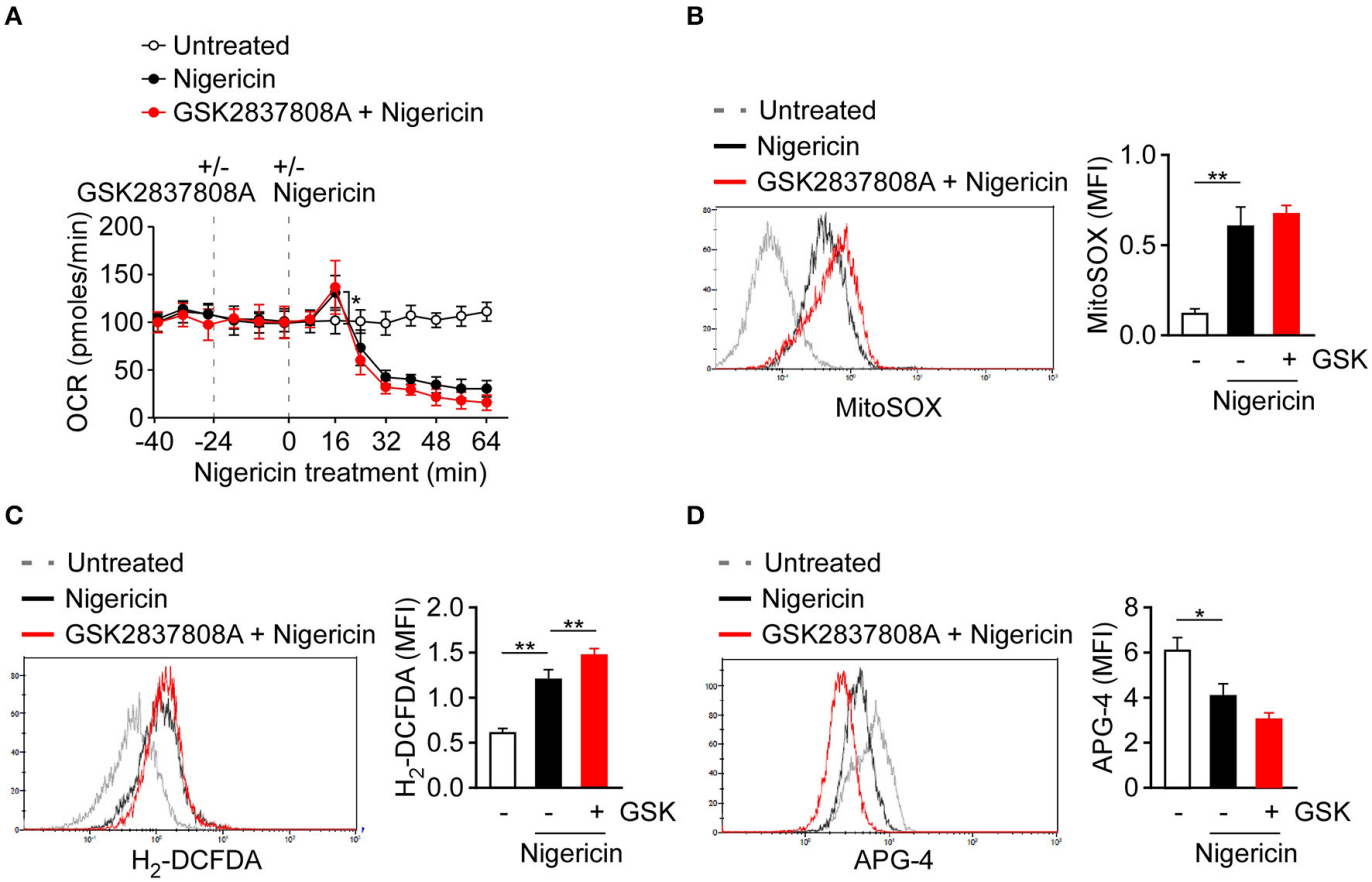
Effect of lactate dehydrogenase inhibition on ROS production and potassium efflux upon NLRP3 inflammasome activation. **(A)** OCR of THP-1-derived macrophages under sequential treatment with or without GSK2837808A and/or nigericin as indicated by dotted vertical lines. **(B, C)** Flow cytometry analysis of mitochondrial ROS production **(B)** and intracellular ROS production **(C)** in THP-1-derived macrophages pretreated with GSK2837808A for 1 h and then stimulated with nigericin for 30 min. **(D)** Flow cytometry analysis of intracellular potassium ion concentration by using APG-4 in THP-1-derived macrophages that had been pretreated with GSK2837808A for 1 h, followed by stimulation with nigericin for 30 min. *P < 0.05; **P < 0.01. All results are presented as the mean ± SD of data from three independent experiments (*n* = 3 per group), and were analyzed with the ANOVA test followed by *post hoc* test.

Production of mtROS, the by-products of mitochondrial respiration, is one of the common signals of NLRP3 inflammasome activation (13–15). As pyruvate is a key metabolite that feeds into mitochondria, we assessed if pyruvate oxidation instead for lactic acid fermentation affected NLRP3 inflammasome through respiration and ROS production. To examine the change of lactic acid fermentation in activation of respiration, we inhibited lactic acid fermentation in macrophages using GSK2837808A. Mitochondrial respiration rate as reflected by OCR was activated in nigericin-treated cells, compared with untreated control cells, but was not affected by pretreatment with GSK2837808A (Figure 4A). Next, we examined whether lactic acid fermentation might affect production of mtROS or cellular ROS in THP-1-derived macrophages. In response to the NLRP3 activator, nigericin, the levels of mtROS (Figure 4B) and intracellular ROS (Figure 4C) were increased. However, unexpectedly, production of neither mtROS nor intracellular ROS was preventable by GSK2837808A pretreatment. In addition to ROS production, potassium efflux is a common trigger of NLRP3 inflammasome activation (4, 13). We examined whether lactic acid fermentation might affect potassium efflux in THP-1-derived macrophages. In response to nigericin stimulation, the levels of intracellular potassium ion were decreased, which was not preventable by GSK2837808A pretreatment (Figure 4D).

### Inhibition of Lactic Acid Fermentation Protects Mice From MSU-Induced Peritonitis

**Figure 5:**
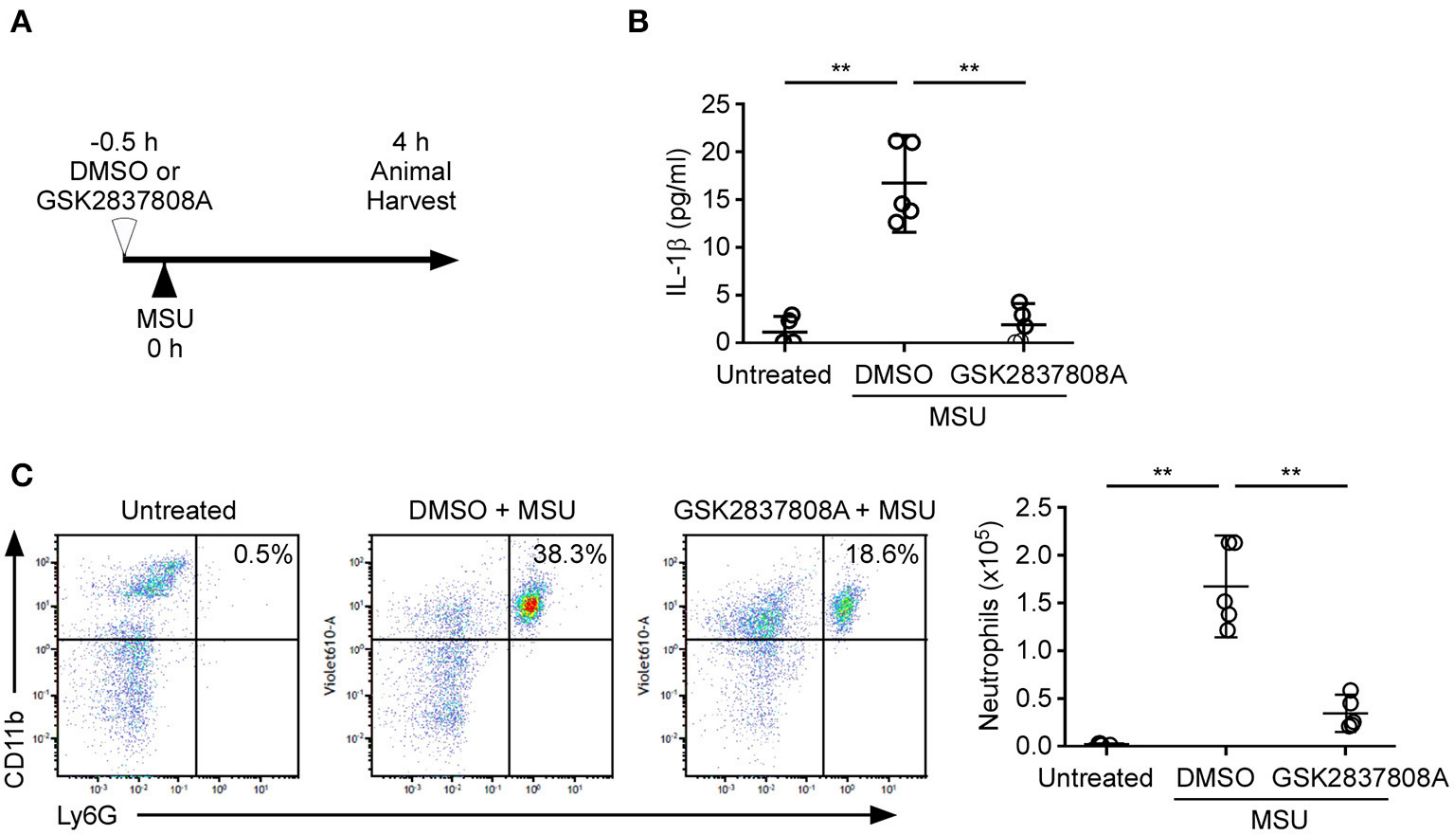
Protective effect of GSK2837808A on MSU-induced peritonitis. **(A)** Schematic presentation of MSU-induced peritonitis in mice. **(B)** Determination by ELISA of IL-1β in the peritonea of mice treated for 4 h with intraperitoneal injection of MSU in the presence or absence of GSK2837808A. **(C)** Absolute number of CD45^+^/CD11b^+^/Ly6G^+^ neutrophils in the peritonea of mice treated for 4 h with intraperitoneal injection of MSU in the presence or absence of GSK2837808A. Each symbol represents an individual mouse (*n* = 5 per group); small horizontal lines indicate the mean ± SD of the data from two independent experiments. All results were analyzed with the ANOVA test followed by *post hoc* test. **P < 0.01.

To determine the biological significance of lactic acid fermentation effects on NLRP3 inflammasome activation *in vivo*, we analyzed an indicator of inflammation – the recruitment of inflammatory cells into the peritoneal cavity—following intraperitoneal injection of mice with MSU (Figure 5A) (28). We examined if treatment with GSK2837808A can inhibit MSU-induced NLRP3 inflammasome activation *in vitro* (Figure 1C) and *in vivo*. MSU induced the production of IL-1β (Figure 5B) and the recruitment of neutrophils (Figure 5C) to the peritoneal cavity, which was efficiently inhibited by GSK2837808A pretreatment.

## Discussion

**Figure 6:**
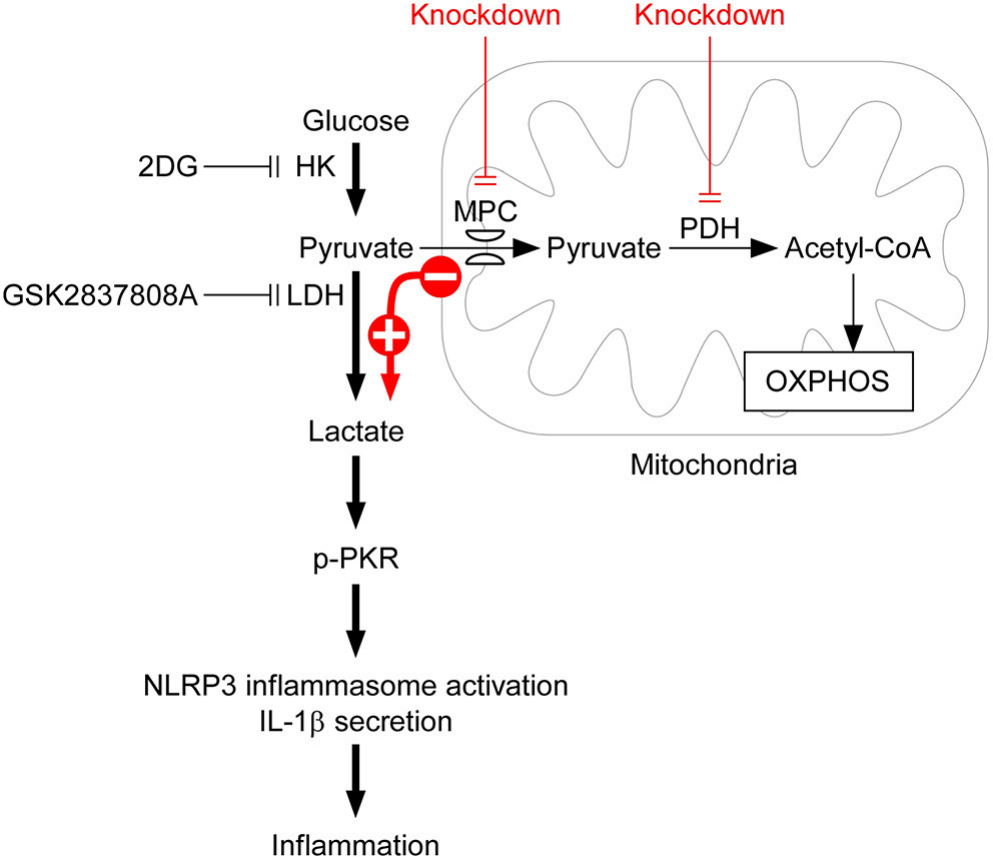
Model for lactic acid fermentation-dependent activation of the NLRP3 inflammasome. Upon stimulation with NLRP3 inflammasome agonist, HK-dependent glycolysis is activated and contributes to NLRP3 inflammasome activation. Based on the results obtained in this study, we propose that lactic acid fermentation following glycolytic flux is required for NLRP3 inflammasome activation, whereas pyruvate oxidation is not. By contrast, downregulation of lactic acid fermentation by 2DG or GSK2837808A inhibits lactate-dependent phosphorylation of PKR and consequently prevents NLRP3 inflammasome activation, IL-1β secretion, and inflammation. Although pyruvate oxidation converts pyruvate into acetyl-CoA, which is important for fueling respiration, inhibition of pyruvate oxidation by depletion of either MPC or PDH reprograms pyruvate metabolism from mitochondrial pyruvate-driven respiration to cytoplasmic lactic acid fermentation, and subsequently enhances activation of the NLRP3 inflammasome.

Activation of the NLRP3 inflammasomes is critical for immune defense. However, improper and excessive activation of NLRP3 inflammasome can lead to inflammatory disease. Much remains to be learned regarding the mechanisms that restrain inflammasome activation under normal conditions. Based on the results obtained in this study, we have proposed a mechanism of lactic acid fermentation-dependent activation of the NLRP3 inflammasome (Figure 6). In response to NLRP3 inflammasome agonist stimulation, lactic acid fermentation is increased and contributes to lactate production, which induces PKR phosphorylation and thereby activates the NLRP3 inflammasome. In addition, pyruvate oxidation also plays a role in NLRP3 inflammasome activation. Inhibition of pyruvate oxidation reprograms pyruvate metabolism from mitochondrial pyruvate-driven respiration to cytoplasmic lactic acid fermentation and thus enhances NLRP3 inflammasome activation.

Previously, we revealed that Cbl negatively regulates NLRP3 inflammasome activation through inhibition of phosphorylated Pyk2 (p-Pyk2)-regulated ASC oligomerization (21, 29) and inhibition of glucose transporter 1 (GLUT1)-regulated glucose utilization (17). Cbl reduces the level of p-Pyk2 through ubiquitination-mediated proteasomal degradation. Since p-Pyk2-phosphorylated ASC is required for ASC oligomerization, downregulation of p-Pyk2 inhibits NLRP3 inflammasome activation (29). In addition to p-Pyk2, Cbl reduces the level of GLUT1, which contributes to glucose uptake and increased glucose utilization in macrophages (30). Inhibiting Cbl increases the surface expression of GLUT1 protein, which increases cellular glucose uptake and consequently raises glycolytic capacity, and in turn enhances NLRP3 inflammasome activation (17). In this study, we further showed that lactic acid fermentation following glycolysis was required for NLRP3 inflammasome activation (Figure 1). Since phosphorylation of PKR was required for NLRP3 inflammasome activation (20), lactate-dependent PKR phosphorylation was responsible for glycolysis-regulated activation of the NLRP3 inflammasome (Figure 2E), but mtROS production was not sufficient (Figure 4B). Consistent with our findings, lactate treatment was shown to increase the expression level of phosphorylated PKR (19). In addition to lactic acid fermentation, pyruvate-driven respiration was activated upon NLRP3 inflammasome activation and it may contribute to mtROS production (17). MtROS is one of the common triggers of the NLRP3 inflammasome (13). Downregulation of glycolysis by HK1 inhibition inhibited mtROS production (18), suggesting that pyruvate oxidation or pyruvate-driven respiration may contribute to NLRP3 inflammasome activation. Thus, unexpectedly, downregulation of mitochondrial pyruvate transport or pyruvate oxidation resulted in an increase in NLRP3 inflammasome activation (Figures 3B,C), which was accompanied by an increase in lactic acid fermentation (Figure 3D). Consistent with our findings, genetic inhibition of MPC1 or MPC2 resulted in a phenotype consistent with a defect in pyruvate transport including lactic acidosis and diminished acetyl-CoA formation and pyruvate-driven mitochondrial respiration (31–33). Inhibition of pyruvate oxidation reprogrammed pyruvate metabolism from mitochondrial pyruvate-driven respiration to cytoplasmic lactic acid fermentation and further activates the regulator of NLRP3 inflammasome activation, lactate-dependent PKR phosphorylation. Together, the findings of this study support that Cbl is a master regulator of NLRP3 inflammasomes. Cbl can inhibit the NLRP3 inflammasomes through two pathways, by inhibition of p-Pyk2-regulated ASC oligomerization and inhibition of GLUT1-regulated lactic acid fermentation.

Increasing evidence suggests that improper activation of the NLRP3 inflammasome is responsible for the pathogenesis of several glucose metabolism-associated diseases such as type 2 diabetes (6) and cancer (12). Diabetes is one of an array of metabolic disorders characterized by high blood glucose. The NLRP3 inflammasome was suggested to play an important role in the pathogenesis of type 2 diabetes, since NLRP3 knockout mice showed improved glucose tolerance and insulin sensitivity (34). High glucose concentration induced NLRP3-dependent IL-1β secretion from pancreatic islet cells, suggesting that it may cause NLRP3 inflammasome activation in islet cells, which leads to the loss of insulin-producing islet cells through pyroptotic cell death and finally contributes to the pathogenesis of diabetes (34). In addition, upregulated NLRP3 inflammasome activation was observed in macrophages from patients with type 2 diabetes (6). Consistent with previous studies, our findings have substantiated the notion that glucose metabolism is important for activation of the NLRP3 inflammasome.

In addition to diabetes, glycolysis is also important for cancer development. The Warburg effect, a hallmark of cancer cells, exhibits high glucose uptake and excessive lactate formation even when the supply of oxygen is sufficient (35). Increasing evidence shows that the NLRP3 inflammasome is involved in carcinogenesis of breast, colon, lung, skin, prostate, pancreas, and stomach cancers (36). Our previous study showed that the NLRP3 inflammasome is highly expressed in nasopharyngeal carcinoma (23) and oral cavity squamous cell carcinoma (37). In a mouse model of breast cancer, either knockout of NLRP3, caspase-1, or IL-1β, or blocking of IL-1β reduces tumor growth (38, 39). In a patient with breast cancer, the expression of NLRP3 protein in the tumor-associated macrophages is correlated with lymphangiogenesis and metastasis (40). Since the Warburg effect is a hallmark of cancer, lactic acid fermentation in cancer cells probably contributes to carcinogenesis through NLRP3 inflammasome activation. In addition, lactic acid is exported into the extracellular environment via monocarboxylate transporters (MCTs) (41), which strongly inhibit T cell mediated anti-tumor immune responses (42, 43). Inhibition of lactic acid fermentation may inhibit tumor growth through inhibition of the NLRP3 inflammasome activation or restoration of T cell-mediated antitumor immunity. In agreement with our suggestion, GSK2837808A treatment inhibited tumor growth in hypoxic conditions (44) and thus improved the efficacy of adoptive T cell therapy (45).

In summary, we propose a model for lactic acid fermentation-dependent activation of the NLRP3 inflammasome. We discovered that lactic acid fermentation following glycolytic flux is important for NLRP3 inflammasome activation, but pyruvate oxidation pathway following glycolytic flux is not. Lactate-dependent PKR phosphorylation may thus be responsible for the observation that glycolysis regulates NLRP3 inflammasome activation. Inhibition of pyruvate oxidation reprograms pyruvate metabolism from pyruvate-driven mitochondrial respiration to cytoplasmic lactic acid fermentation and consequently enhances NLRP3 inflammasome activation. Reprograming pyruvate metabolism in mitochondria and in the cytoplasm may thus be a novel strategy for treatment of NLRP3 inflammasome -associated inflammatory diseases.

## Data Availability Statement

The original contributions generated for the study are included in the article/supplementary material, further inquiries can be directed to the corresponding author/s.

## Ethics Statement

The animal study was reviewed and approved by The Institutional Animal Care and User Committee of MacKay Medical College.

## Author Contributions

L-CC: conceptualization, visualization, and project administration. K-YH: formal analysis. H-CL, Y-JC, Y-HW, and L-CC: funding acquisition. H-CL, Y-JC, H-AL, K-HL, H-HW, and T-FL: investigation. C-CC: methodology. Y-LH: validation. H-CL and L-CC: writing — original draft. Y-HW: writing, review and editing.

## Conflict of Interest

The authors declare that the research was conducted in the absence of any commercial or financial relationships that could be construed as a potential conflict of interest.
